# Individual and joint effects of overweight/obesity and the triglyceride−glucose index on mortality risk in type 2 diabetic patients: a retrospective cohort study in China

**DOI:** 10.3389/fendo.2026.1652682

**Published:** 2026-02-06

**Authors:** Anthony Diwon, Yuxin Chen, Yang Chen, Xinlv Zhang, Hui Wang, Ziyi Wang, Guomiao Zhang, Qichao Sheng, Huiqin Mei, Yixi Xu, Qingyang Mao, Chao Zheng, Xiaoyu Zhang, Guangyun Mao

**Affiliations:** 1 Department of Epidemiology & Biostatistics, School of Public Health, Wenzhou Medical University, Wenzhou, Zhejiang, China; 2 Department of Endocrinology, The Second Affiliated Hospital, Wenzhou Medical University, Wenzhou, Zhejiang, China; 3 State Key Laboratory of Cognitive Intelligence, University of Science and Technology of China, Hefei, Anhui, China; 4 Department of Endocrinology, The Second Affiliated Hospital, School of Medicine, Zhejiang University, Hangzhou, Zhejiang, China

**Keywords:** mortality risk, obesity, overweight, triglyceride glucose index, type 2 diabetes

## Abstract

**Background:**

The global prevalence of type 2 diabetes mellitus (T2DM) has risen significantly since 1990, contributing substantially to mortality and posing a major public health challenge. While overweight/obesity and insulin resistance, commonly reflected by the triglyceride-glucose (TyG) index, are established risk factors for the development of T2DM, their individual and combined effects on mortality among patients with T2DM remain incompletely elucidated. This study aimed to evaluate the associations between body mass index (BMI) and the TyG index with all-cause and cardiovascular disease (CVD) mortality in a large clinical cohort of type 2 diabetes mellitus (T2DM) patients.

**Methods:**

This retrospective cohort study included 15,796 T2DM adults (aged >18 years) from two hospitals in China (2010-2023). The primary outcome was all-cause mortality, with a mean follow-up duration of 2.8 years. BMI was categorized as normal weight (18.5-24.9 kg/m^2^), overweight (25-29.9 kg/m^2^), and obesity (≥30 kg/m^2^). The TyG index was calculated as ln [fasting triglycerides (mg/dL) × fasting glucose (mg/dL)/2]. Multiple Cox proportional hazards models were used to estimate adjusted hazard ratios (aHRs) and 95% confidence intervals (CIs) for mortality.

**Results:**

Among 15,796 participants, 1,665 deaths were recorded, including 629 CVD-related deaths. Overweight and obesity were associated with lower all-cause mortality risk (aHR: 0.73, 95% CI: 0.65−0.81 and aHR: 0.86, 95% CI: 0.74−1.00, respectively). Higher TyG index quartiles (Q3 and Q4) were associated with decreased mortality risk (aHR: 0.85, 95% CI: 0.74−0.98; aHR: 0.84, 95% CI: 0.72−0.97). The protective effect of a higher BMI was more pronounced in patients aged 60 years or older.

**Conclusion:**

Our findings revealed that BMI and the TyG index are associated with mortality risk in T2DM patients, particularly in older patients. However, these findings are observational and do not imply causality or validate prognostic use. Further studies using causal inference methods are necessary to inform clinical guidelines.

## Background

The prevalence and incidence rates of type 2 diabetes mellitus (T2DM) have risen steadily globally since 1990. Previous studies reported that 529 million people of all ages worldwide have diabetes, the prevalence of diabetes has increased from 3·2% (3·0–3·5) to 6·1% in 2021, and the prevalence of diabetes has increased by 59.7% (95% CI: 54·7–66·0) from 6.1% (5·8–6·5) to 9·8% (9·4–10·2), resulting in 1.31 billion (1·22–1·39) people living with diabetes in 2050 (1). T2DM and cardio-cerebrovascular disease (CCVD) account for more than 80% of all premature noncommunicable disease (NCD)-related deaths (2, 3). Consequently, T2DM has emerged as a significant global public health challenge, as it is a major contributor to mortality and morbidity, reduces life expectancy, and hinders socioeconomic progress (4, 5). A previous study reported that 1 in every 11 people, 366 million people, or 28% of the total global population, have type 2 diabetes. This incidence peaks at age 55 with an equal sex distribution and is a significant cause of death worldwide, with approximately 1 million fatalities annually in the past three decades (4, 6). Importantly, diabetes mellitus, particularly T2DM, is linked to a markedly increased risk of mortality, accounting for 6.7 million diabetes-related deaths worldwide; in China, T2DM-related deaths account for more than 3.9% of all deaths (7, 8).

Overweight and obesity are prevalent in individuals with T2DM and are established risk factors for the onset of T2DM (9–12). However, emerging literature has reported counterintuitive findings suggesting that overweight or obese individuals with T2DM may experience lower mortality risk a phenomenon termed the “obesity paradox.” The mechanisms behind this paradox are not well understood and may be attributed to reverse causation (e.g., weight loss due to preexisting illness), collider bias (e.g., medication use, healthcare access), and residual confounding (13, 14). Previous studies have reported a strong correlation between obesity and the development and exacerbation of T2DM (15, 16).

T2DM is a multifactorial metabolic disease characterized by elevated blood sugar levels resulting from inadequate insulin production and resistance to insulin action (9). Insulin resistance is widely accepted to be strongly associated with the prognosis of T2DM patients (10–12). The triglyceride glucose (TyG) index is a simple, accessible, and reliable clinical surrogate marker of insulin resistance that can be easily determined via the formula TyG=ln[Triglycerides(mg/dl)*fasting plasma glucose(mg/dl)/2] It does not depend on insulin measurement and might be applied to all individuals, irrespective of their treatment (10, 13, 14). The TyG index is associated with mortality risk and cardiovascular diseases, including carotid atherosclerosis, coronary artery disease, metabolic syndrome, and type 2 diabetes mellitus (T2DM) [22-24]. However, further studies on the TyG index for mortality risk in type 2 diabetes mellitus (T2DM) patients are needed. The association between this condition and insulin resistance markers, such as the TyG index, and how these combined factors influence mortality remains unclear. This study aimed to investigate the individual and combined effects of overweight/obesity and the triglyceride-glucose (TyG) index on mortality risk in type 2 diabetes mellitus (T2DM) patients.

## Methods

### Study population

**Figure 1:**
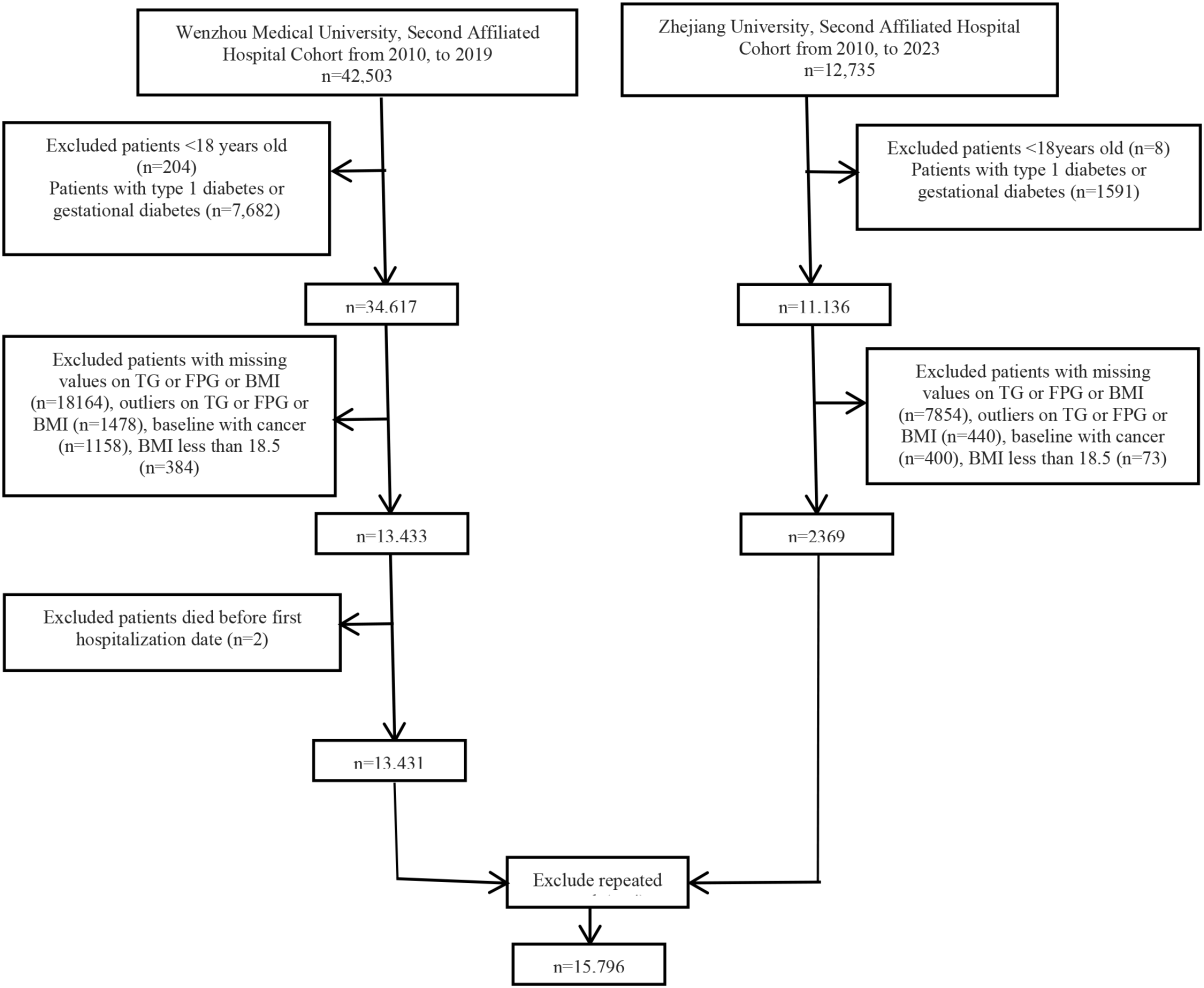
Flow chart for study participants.

This two-center, retrospective cohort study extracted data from the electronic medical records (EMRs) of two premier hospitals in Zhejiang Province, P.R. China, rather than relying on self-report questionnaires. These two centers included the Second Affiliated Hospital of Wenzhou Medical University from January 1, 2010, to December 31, 2019, and the Second Affiliated Hospital of Zhejiang University, spanning from January 1, 2010, to July 1, 2023. A total of 55,502 participants (n=42,503 and n=12,735 from the Wenzhou and Hangzhou cohorts, respectively) diagnosed with type 2 diabetes were included in the cohort. The exclusion criteria included (1) individuals aged less than 18 years; (2) those with type 1 diabetes, gestational diabetes, or other specific diabetes; (3) absence of BMI, TG, or FPG; (4) BMI less than 18.5; (5) outliers of BMI, TG or FPG, values below 2% and above the 98% quantile; (6) individuals with cancer; (7) individuals who died before admission; and (8) individuals whose records were duplicated. Experienced physicians meticulously assessed the baseline characteristics of the individuals during their initial hospital stay. Finally, the data analysis included 15,796 patients with T2DM (**Figure 1**).

### Data collection

Data related to social and clinical aspects were retrieved from electronic medical records (EMRs) via a structured questionnaire explicitly created for this study. The collected information included demographic details (sex, age, etc.) and anthropometric measures (height, weight, waist circumference, BMI, etc.). The medical history of patients included triglyceride (TG), fasting plasma glucose (FPG), hypertension, stroke, and other comorbidities; lifestyle history (alcohol use, smoking status, etc.); laboratory results of cholesterol (HDL, LDL) levels; fasting blood sugar and hemoglobin (HbAlc) levels; estimated glomerular filtration rate (eGFR, calculated via the CKD-EPI equation) (15); and information about hospitalization-related data (admission and discharge date, duration of stay and total charge). Mortality data were obtained from China’s Population Death Register Information System, using participants’ resident identification card numbers and recorded up to the study endpoints of December 31st, 2019, for the Wenzhou Medical University cohort and July 1st, 2023, for the Zhejiang University cohort. The data were retrieved using the participant’s resident identity card number. Ethical approval for the study was obtained and informed consent was waived by the institutional review board because of the retrospective nature of the study.

### Definition of exposure and clinical outcomes

The Chinese Center for Disease Control and Prevention defines body mass index(BMI) as <18.5 kg/m2 (underweight), 18.5−23.9 kg/m2 (normal weight), 24−27.9 kg/m2 (overweight), and >28 kg/m2 (obesity); a normal BMI among healthy Chinese adults (18–64 years old) ranging from 18.5 kg/m2 to 23.9 kg/m2 and a BMI >24 kg/m2 indicate that the individual is overweight and >28 kg/m2 indicates obesity (16–18). The triglyceride glucose (TyG) index is a simple, cost-effective surrogate marker of glycemic control and insulin resistance in individuals with T2DM. The normal range was established from 4–8, with values exceeding 8 indicating an elevated risk (10, 19, 20).

The primary outcome of the study was all-cause mortality. We focused on mortality from all causes as well as cause-specific deaths, such as those caused by heart disease and other conditions. The causes of death were categorized via the International Classification of Diseases, 10th revision (ICD-10). CCVD mortality was recorded under codes I00-I9, I11, I13, I20-I51, and I60-I69. Our primary focus was all-cause mortality, including codes representing deaths from different causes. A more thorough definition was also given by the secondary outcomes, which further divided CCVD death into categories: cerebrovascular illnesses (160-69), ischemic heart diseases (IHDs, 120-125), hypertensive heart disorders (111), and other kinds of CCVD mortality.

### Statistical analysis

Thorough data preprocessing was performed with rigorous methods to minimize potential bias and guarantee the quality and dependability of our results. A value below 2.5% or above 97.5% was considered an outlier in the covariates and was considered missing. Similarly, data that fell outside the range of the mean +/- 2.5 times the standard deviation were likewise deemed missing. If the missing data did not exceed 30% and had a random pattern, the missing values were imputed via a 5-fold multiple imputation (MI) method based on the Markov chain Monte Carlo (MCMC) methodology. The first quartile and third quartile of the median for continuous variables and the proportion of frequency for categorical variables were used to represent the baseline characteristics. The Wilcoxon rank sum test was used to determine how the continuous variables differed between the living and dead groups, and the chi-square test was used to compare categorical data between groups. Furthermore, to mitigate the bias resulting from the large sample size, the standardized mean difference (SMD) was used to highlight the differences between the two groups. Disagreements with SMD > 0.1 were deemed to reach a substantial threshold. The independent effects of BMI and TyG level on the outcomes were thoroughly examined via Cox proportional hazard models. Covariates were absent from the first model (univariate). The adjusted model modified the effects of sex, age, alcohol use, smoking status, hypertension, coronary heart disease (CHD), stroke, fatty liver disease (FLD), glycosylated hemoglobin A1c (HbA1c), high-density lipoprotein cholesterol (HDL-C), low-density lipoprotein cholesterol (LDL-C) and estimated glomerular filtration rate (eGFR). Considering TyG as a continuous variable [scaled to the interquartile range (IQR)] and as a categorical variable, adjusted hazard ratios (aHRs) and 95% confidence intervals (CIs) were further calculated to quantify the effects of the TyG Index on mortality. The possible dose-response connections between TyG levels and all-cause mortality were first assessed using restricted cubic spline (RCS) regression models, which incorporate covariates from the adjusted model and position knots at the 10th, 50th, and 90th percentiles. Nonlinearity was assessed via a likelihood ratio test. The joint effects of TyG and BMI were also evaluated using Cox proportional hazard regression in both crude and adjusted models, which were employed to assess the associations. For models of the joint effects of TyG and BMI, TyG was a binary variable, with high exposure defined as exceeding the median exposure for the exposure period, and BMI was a three-level categorical variable (normal weight, overweight, and obesity), with normal weight serving as the reference category. The TyG index was dichotomized in the joint analyses using the sample median (TyG =9.08) due to the absence of a universally established clinical cutoff for mortality risk stratification in patients with T2DM. This median-based approach ensured balanced group sizes across exposure categories, improving the stability of statistical estimates and the interpretability of interaction effects. Furthermore, exploratory analyses using restricted cubic splines revealed an approximately linear negative association between the TyG index and mortality risk, supporting the use of a binary categorization scheme for analytical simplicity without substantial loss of information. Two product interaction terms, TyG with overweight and TyG with obesity, were used to evaluate interactions on a multiplicative scale. In addition, the additivity of effects was evaluated via the dance estimate recovery (MOVER) method. A RERI of 0 indicates no interaction.

A two−tailed p value of less than 005 or a standardized mean difference (SMD) greater than 0.1 was used to assess statistical significance. All data management and analysis were performed via R.3.0 (Copyright ^©^ 2024 The R Foundation for Statistical Computing).

### Sensitivity analysis

Furthermore, we conducted several sensitivity analyses to ensure the reliability of our results. First, we compared the data sets before and after imputation to assess the imputation effect. Second, to address the potential for reverse causation due to severe disease and decreased survival bias, patients who died within two years of follow-up were excluded. Furthermore, considering the competing risk factors for cause-specific mortality, the Fine-Grey model was used to assess the associations between the SII and CCVD mortality. Finally, the individual and joint effects of TyG and BMI were stratified by age.

## Results

The retrospective cohort study included 15,796 T2DM patients aged 18 years and older, with a median follow-up duration of 3.8 years. Among the total population, 53.4% were males, and the total number of participants who were overweight or obese was 54.7% (BMI 24.0–27.9 kg/m²) was 6,410, 40.6% (BMI ≥28.0 kg/m²), was 2,234(14.1%) (**Table 1**). **Table 2** shows a detailed comparison of the baseline characteristics of deceased participants and their counterparts with T2DM according to all-cause mortality. The total number of deaths observed among the participants was 1,665, and the TyG level was stratified into quartiles (Q1-Q4). In Q1 (TyG 7.46-8.67), a mortality rate of 12.2% was reported, which was higher than that in Q2-Q4. Age: Deceased participants were generally older than living participants (SMD = 0.798), with a significant association between stroke incidence and mortality (SMD = 0.266), a higher rate of hypertension in the deceased groups (0.201), higher mortality rates among individuals with fatty liver disease (0.328) and lower kidney function (SMD = 0.521). The differences in many covariates between the two groups are quite significant, indicating that these profiles may be potential confounders when examining the associations between overweight/obesity and the TyG index with mortality risk among T2D individuals.

**Table 1:** Individual effects of BMI and the TyG index on the risk of mortality in patients with type 2 diabetes.

Variable	N	Death Number (%)	Crude		Adjusted	
			HR (95% CI)	*P* value	HR (95% CI)	*P* value
All-cause mortality						
BMI, kg/m^2^						
18.5-23.9 (Normal weight)	7152	882 (12.3)	Reference		Reference	
24.0-27.9 (Overweight)	6410	568 (8.9)	0.72 (0.65, 0.80)	<0.001	0.73 (0.65, 0.81)	<0.001
≥28.0 (Obesity)	2234	215 (9.6)	0.79 (0.68, 0.91)	0.002	0.86 (0.74, 1.00)	0.056
Per kg/m^2^			0.96 (0.94, 0.97)	<0.001	0.97 (0.95, 0.98)	<0.001
TyG						
Q1 (7.46-8.67)	3949	481 (12.2)	Reference		Reference	
Q2 (8.67-9.08)	3949	414 (10.5)	0.86 (0.76, 0.99)	0.030	0.93 (0.81, 1.06)	0.262
Q3 (9.08-9.52)	3949	389 (9.9)	0.80 (0.70, 0.91)	0.001	0.85 (0.74, 0.98)	0.021
Q4 (9.52-11.1)	3949	381 (9.7)	0.78 (0.68, 0.89)	<0.001	0.84 (0.72, 0.97)	0.015
Per IQR = 0.85			0.88 (0.83, 0.94)	<0.001	0.91 (0.85, 0.97)	0.005
CCVD mortality						
BMI, kg/m^2^						
18.5-23.9 (Normal weight)	7152	378 (5.3)	Reference		Reference	
24.0-27.9 (Overweight)	6410	240 (3.7)	0.71 (0.60, 0.84)	<0.001	0.69 (0.58, 0.81)	<0.001
≥28.0 (Obesity)	2234	111 (5.0)	0.95 (0.77, 1.17)	0.618	1.03 (0.83, 1.28)	0.789
Per kg/m^2^			0.98 (0.96, 1.00)	0.060	0.99 (0.96, 1.01)	0.201
TyG						
Q1 (7.46-8.67)	3949	205 (5.2)	Reference		Reference	
Q2 (8.67-9.08)	3949	195 (4.9)	0.96 (0.79, 1.16)	0.650	1.00 (0.81, 1.22)	0.972
Q3 (9.08-9.52)	3949	160 (4.1)	0.77 (0.63, 0.95)	0.013	0.77 (0.62, 0.96)	0.018
Q4 (9.52-11.1)	3949	169 (4.3)	0.81 (0.66, 0.99)	0.041	0.81 (0.65, 1.01)	0.064
Per IQR = 0.85			0.88 (0.80, 0.97)	0.007	0.87 (0.79, 0.96)	0.007

BMI, body mass index; TyG, triglyceride−glucose; HR, hazard ratio; CI, confidence interval, CCVD, cardio-cerebrovascular diseases; RERI, relative excess risk due to interaction. Adjusted for age, sex, hypertension, coronary heart disease, stroke, fatty liver disease, smoke, drink, glycosylated hemoglobin A1c, high-density lipoprotein cholesterol, low-density lipoprotein cholesterol, and estimated glomerular filtration rate.

**Table 2:** Baseline demographic and clinical characteristics of patients with type 2 diabetes according to all-cause mortality.

Variables	Overall (n=15796)	Alive (n=14131)	Dead (n=1665)	P value	SMD
Sex				0.014	0.064
Male	8433 (53.4)	7497 (53.1)	936 (56.2)		
Female	7363 (46.6)	6634 (46.9)	729 (43.8)		
Smoke				0.893	0.003
No	11828 (74.9)	10579 (74.9)	1249 (75.0)		
Yes	3968 (25.1)	3552 (25.1)	416 (25.0)		
Drink				0.050	0.052
No	12367 (78.6)	11034 (78.4)	1333 (80.4)		
Yes	3372 (21.4)	3048 (21.6)	324 (19.6)		
CHD				0.001	0.083
No	5192 (32.9)	4586 (32.5)	606 (36.4)		
Yes	10604 (67.1)	9545 (67.5)	1059 (63.6)		
Stroke				<0.001	0.266
No	12620 (79.9)	11459 (81.1)	1161 (69.7)		
Yes	3176 (20.1)	2672 (18.9)	504 (30.3)		
Hypertension				<0.001	0.201
No	10774 (68.2)	9504 (67.3)	1270 (76.3)		
Yes	5022 (31.2)	4627 (32.7)	395 (23.7)		
Fatty liver disease				<0.001	0.328
No	6564 (41.6)	6103 (43.2)	461 (27.7)		
Yes	9232 (58.5)	8028 (56.8)	1204 (72.3)		
Age, years	65.0 (57.0, 73.0)	64.0 (57.0, 72.0)	74.0 (65.0, 80.0)	<0.001	0.798
HDL-C, mmol/L	1.0 (0.9, 1.2)	1.0 (0.9, 1.2)	0.9 (0.8, 1.1)	<0.001	0.157
LDL-C, mmol/L	2.5 (1.9, 3.2)	2.5 (1.9, 3.2)	2.4 (1.8, 3.1)	<0.001	0.108
SCr, μmol/L	64.7 (53.5, 79.0)	63.7 (53.0, 77.5)	73.9 (60.1, 93.1)	<0.001	0.471
HbA1c, %	7.8 (6.9, 8.8)	7.8 (6.9, 8.8)	7.9 (7.0, 8.9)	0.006	0.062
FPG, mmol/L	6.9 (5.7, 8.8)	6.9 (5.7, 8.8)	7.1 (5.6, 9.2)	0.095	0.064
TG, mmol/L	1.5 (1.1, 2.2)	1.6 (1.1, 2.2)	1.4 (1.0, 2.0)	<0.001	0.136
eGFR (mL/min per 1.73 m, %)	94.9 (76.6, 115.1)	96.8 (78.6, 116.5)	79.8 (62.8, 99.1)	<0.001	0.521

Normally distributed continuous variables are described as the means ± standard deviations, and continuous variables without a normal distribution are presented as medians (1st quartile, 3rd quartile). Categorical variables are presented as numbers (percentages). CHD, coronary heart disease; HDL-C, high-density lipoprotein cholesterol; LDL-C, low-density lipoprotein cholesterol; SCr, serum creatinine; HbA1c, glycosylated hemoglobin A1c; FPG, fasting plasma glucose; TG, triglyceride; eGFR, estimated glomerular filtration rate; SMD, standardized mean difference.

### Individual effects of BMI and TyG on the risk of mortality in patients with type 2 diabetes

**Figure 2:**
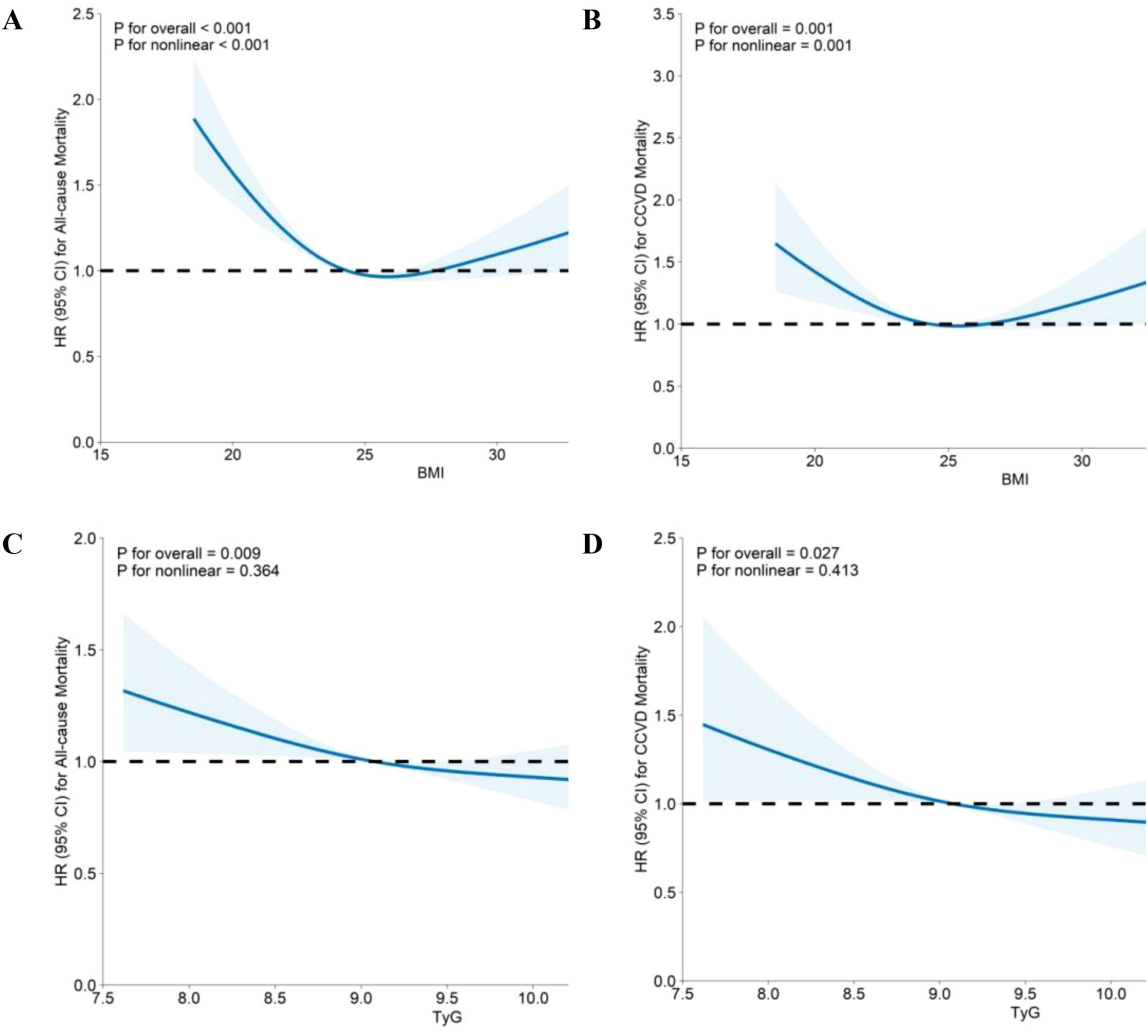
Associations of BMI and the TyG index with the risk of all-cause mortality and CCVD mortality based on spline regression models. BMI, body mass index; TyG, triglyceride−glucose; HR, hazard ratio; CI, confidence interval; CCVD, cardio-cerebrovascular disease. Age, sex, TyG, hypertension, coronary heart disease, stroke, fatty liver disease, smoking, drinking, glycosylated hemoglobin A1c, high-density lipoprotein cholesterol, low-density lipoprotein cholesterol, and estimated glomerular filtration rate were adjusted for in the association between BMI and mortality. Age, sex, BMI, hypertension, coronary heart disease, stroke, fatty liver disease, smoking, drinking, glycosylated hemoglobin A1c, high-density lipoprotein cholesterol, low-density lipoprotein cholesterol, and estimated glomerular filtration rate were adjusted for the associations between TyG and mortality. **(A)** BMI and all-cause mortality; **(B)** BMI and CCVD mortality; **(C)** TyG score and all-cause mortality; **(D)** TyG score and CCVD mortality.

The study assessed the impact of BMI and the TyG index on all-cause and CCVD mortality (**Table 1****).** Compared with normal weight individuals, overweight individuals had a lower risk of death (aHR: 0.73, 95% CI: 0.65−0.81), as obesity was associated with a marginally lower risk of all-cause mortality (aHR: 0.86, 95% CI: 0.74−1.00). For the TyG index, those in Q3 and Q4 presented lower mortality risks (p=0.021, p=0.015), whereas those in Q2 were not significantly different from those in the reference group (Q1). Overweight individuals also had a lower risk of CCVD mortality (HR: 0.69), with Q3 TyG participants having an aHR of 0.77. A U-shaped relationship was noted for BMI with all-cause and CCVD mortality risk (nonlinear p<0.001), whereas the TyG index demonstrated a linear decline in mortality risk (nonlinear=0.413) (**Figure 2**).

### Joint effects of BMI and TyG index on the risk of mortality in patients with type 2 diabetes

**Figure 3:**
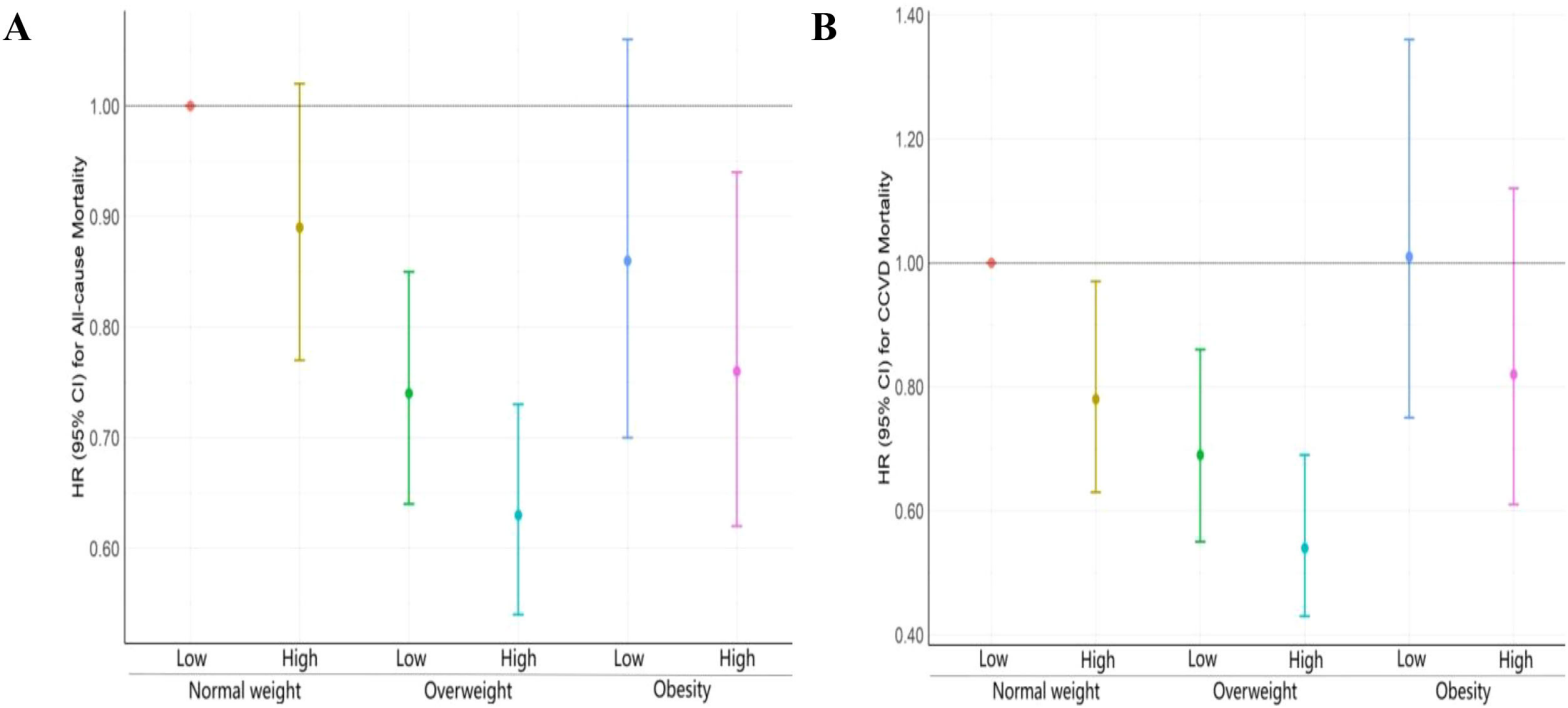
Adjusted combined effects of BMI and the TyG index on the risk of all-cause and CCVD mortality. TyG, triglyceride-glucose; HR, hazard ratio; CI, confidence interval; CCVD, cardio-cerebrovascular disease. Age, sex, body mass index, hypertension, coronary heart disease, stroke, fatty liver disease, smoking, alcohol consumption, glycosylated hemoglobin A1c, high-density lipoprotein cholesterol, low-density lipoprotein cholesterol, and estimated glomerular filtration rate were adjusted for. The normal weight, overweight, and obesity categories indicate BMIs of 18.5–23.9, 24.0–27.9, and ≥28 kg/m2, respectively; the low TyG indicates TyG < 9.08; and the high TyG indicates TyG ≥ 9.08, **(A)** Adjusted combined effects of BMI and TyG index on all-cause mortality risk **(B)** and Adjusted combined effects of BMI and TyG index on CCVD mortality risk.

Furthermore, the joint effect analysis revealed that higher triglyceride levels (TyG ≥9.08) in overweight individuals are associated with a slightly lower risk of all-cause mortality than lower TyG levels are (aHR: 0.63, p<0.01). However, obese individuals with elevated TyG values had decreased risk (aHR: 0.76, p=0.012). Hence, for cardiovascular disease mortality (CCVD), overweight individuals with elevated TyG values presented the lowest risk (aHR 0.54, p<0.001), and obese individuals with elevated TyG values presented a trend similar to that of all-cause mortality. Overall, overweight T2D patients presented a lower all-cause and CCVD-related mortality risk than did the other subgroup of participants did, indicating that the joint effect was more evident in overweight T2D patients (**Table 3**, **Figure 3**).

**Table 3:** Joint effects of BMI and TyG index on the risk of mortality in patients with type 2 diabetes.

BMI, kg/m^2^	TyG≥9.08	N	Dead Number (%)	Crude		Adjusted	
				HR (95% CI)	*P* value	HR (95% CI)	*P* value
All-cause mortality							
18.5-23.9 (Normal weight)	No	3939	499 (12.7)	Reference		Reference	
18.5-23.9 (Normal weight)	Yes	3213	383 (11.9)	0.90 (0.79, 1.03)	0.141	0.89 (0.77, 1.02)	0.095
24.0-27.9 (Overweight)	No	3005	290 (9.7)	0.76 (0.65, 0.87)	<0.001	0.74 (0.64, 0.85)	<0.001
24.0-27.9 (Overweight)	Yes	3405	278 (8.2)	0.63 (0.55, 0.73)	<0.001	0.63 (0.54, 0.73)	<0.001
≥28.0 (Obesity)	No	954	106 (11.1)	0.85 (0.69, 1.05)	0.138	0.86 (0.70, 1.06)	0.166
≥28.0 (Obesity)	Yes	1280	109 (8.52)	0.67 (0.55, 0.83)	<0.001	0.76 (0.62, 0.94)	0.012
Interaction TyG Overweight				1.08 (0.75, 1.14)	0.459	1.04 (0.78, 1.19)	0.711
Interaction TyG Obesity				1.15 (0.65, 1.18)	0.373	1.00 (0.74, 1.34)	0.974
RERI_overweight_ (95% CI)				-0.03 (-0.20, 0.14)		0.00 (-0.17, 0.17)	
RERI_obesity_ (95% CI)				-0.08 (-0.33, 0.16)		0.01 (-0.24, 0.26)	
CCVD mortality							
18.5-23.9 (Normal weight)	No	3939	220 (5.6)	Reference		Reference	
18.5-23.9 (Normal weight)	Yes	3213	158 (4.9)	0.85 (0.69, 1.04)	0.107	0.78 (0.63, 0.97)	0.023
24.0-27.9 (Overweight)	No	3005	124 (4.1)	0.73 (0.59, 0.91)	0.006	0.69 (0.55, 0.86)	0.001
24.0-27.9 (Overweight)	Yes	3405	116 (3.4)	0.60 (0.48, 0.75)	<0.001	0.54 (0.43, 0.69)	<0.001
≥28.0 (Obesity)	No	954	56 (5.9)	1.02 (0.76, 1.37)	0.881	1.01 (0.75, 1.36)	0.952
≥28.0 (Obesity)	Yes	1280	55 (4.3)	0.77 (0.57, 1.04)	0.084	0.82 (0.61, 1.12)	0.213
Interaction TyG Overweight				1.04 (0.70, 1.33)	0.822	0.99 (0.73, 1.40)	0.942
Interaction TyG Obesity				1.12 (0.58, 1.36)	0.598	0.96 (0.68, 1.60)	0.840
RERI_overweight_ (95% CI)				0.02 (-0.23, 0.27)		0.07 (-0.16, 0.31)	
RERI_obesity_ (95% CI)				-0.10 (-0.49, 0.29)		0.03 (-0.35, 0.42)	

BMI, body mass index; TyG, triglyceride-glucose; HR, hazard ratio; CI, confidence interval CCVD, cardio-cerebrovascular disease; RERI, relative excess risk due to interaction. Adjusted for age, sex, hypertension, coronary heart disease, stroke, fatty liver disease, smoking, drinking, glycosylated hemoglobin A1c, high-density lipoprotein cholesterol, low-density lipoprotein cholesterol, and estimated glomerular filtration rate.

### Sensitivity results

The analysis compared results before and after imputing missing data, revealing no significant changes apart from HbA1c, which showed a notable difference (SMD = 0.006, p<0.001) (**Supplementary Table S1**). This validation is crucial for the integrity of the imputed data. For individuals who died after two years of follow-up, obese and overweight individuals had lower risks of all-cause mortality (aHR: 0.93, 95% CI: 0.76−1.13) and obesity (aHR: 0.98, 95% CI: 0.96−1.00) than normal-weight individuals did, while the TyG index showed no significant associations. Compared with overweight and obese individuals, normal-weight individuals had significantly greater CCVD mortality (p=0.003) (**Supplementary Table S2**). When the joint effects for two-year mortality were adjusted, overweight individuals with high TyG indices presented a lower all-cause mortality risk (aHR: 0.70, p<0.001), whereas high TyG indices in obese individuals were not significantly correlated. For CCVD mortality, a high TyG index had protective effects on overweight individuals (aHR: 0.56, p<0.001), whereas the risk among obese individuals remained unchanged (**Supplementary Table S3**). The fine−gray model used to address competing risks in survival analysis revealed that overweight significantly reduced the risk of CCVD mortality compared with that of normal-weight individuals (aHR: 0.70, 95%CI: 0.6−0.083, p<0.001). In contrast, obesity had no significant effect on CCVD mortality risk (aHR: 1.05, 95% CI: 0.85−1.3, P = 0.633). Each increase in BMI of 1 kg/m² was associated with a minor, nonsignificant reduction in CCVD mortality risk (aHR: 0.98, 95% CI: 0.96−1.01, P = 0.128). Notably, TyG levels in the third quartile (Q3, TyG 9.08−9.52) were associated with a significantly lower CCVD mortality risk than those in the first quartile (Q1, TyG 7.46−8.67) with an adjusted HR of 0.78 (95% CI: 0.63−0.97, p=0.026). However, the highest quartile (Q4, TyG 9.52−11.1) did not reveal significant changes in CCVD mortality risk even after adjustment. Each increase in one interquartile range (IQR) of TyG significantly decreased CCVD mortality risk (aHR: 0.88, P = 0.011) (**Supplementary Table S4**). The joint effects of BMI and TyG indicate that overweight individuals with a high TyG index have a significantly reduced CCVD mortality risk (aHR: 0.56 95% CI: 0.45−0.71, p<0.001), and both the low TyG and high TyG groups show a significant reduction in CCVD mortality risk compared with normal weight individuals. The obese individuals revealed no significant difference in CCVD mortality risk irrespective of their TyG index before and after adjustment (aHR=0.85, 95% CI: 0.63−1.15, p=0.281) (**Supplementary Table S5**)). Among patients under 60 years of age, overweight was associated with a significantly lower risk of all-cause mortality compared to normal weight (aHR: 0.58; 95% CI: 0.42–0.80; p = 0.001). In contrast, individuals with obesity showed only a moderate, non-significant risk reduction (aHR: 0.77; 95% CI: 0.51–1.17; p = 0.229), suggesting lower mortality risk among the overweight group. Regarding the TyG index, mortality risk across quartiles Q2 to Q4 did not exhibit a clear trend, with adjusted hazard ratios (aHRs) of 0.92 (p = 0.691), 1.15 (p = 0.493), and 0.87 (p = 0.507), respectively.

A similar pattern was observed for cardio-cerebrovascular disease (CCVD) mortality: overweight significantly reduced mortality risk (aHR = 0.47; 95% CI: 0.26–0.87; p = 0.016), while obesity was not significantly associated with CCVD mortality (aHR = 0.07; 95% CI: 0.58–2.00; p = 0.824).

Among participants aged 60 years and above, a higher body mass index (BMI) was significantly associated with a reduced risk of all-cause mortality. Specifically, overweight conferred a survival advantage (aHR: 0.71, 95% CI: 0.63-0.79), and obesity was also linked to lower mortality (aHR: 0.81, 95% CI: 0.69-0.94). Similarly, for cardio-cerebrovascular disease (CCVD) mortality, overweight individuals experienced a significantly lower risk (aHR: 0.67; 95% CI: 0.57-0.79) compared to those with obesity. In addition, a higher triglyceride-glucose (TyG) index exhibited a reduce association with CCVD mortality, as evidenced in the third (Q_3_: aHR 0.74; 95% CI: 0.60-0.92; p = 0.007) and fourth quartiles (Q_4_: aHR 0.75; 95% CI: 0.60-0.94; p = 0.011) (**Supplementary Table S6**). The joint analysis revealed that in individuals aged < 60 years, overweight individuals with high TyG index face increased mortality risk (HR = 0.59, p=0.021), whereas obese individuals show protective trends. In older adults, overweight and obese individuals with high TyG indices had significantly increased all-cause mortality risk (aHR=0.58 and HR = 0.67, respectively), whereas obesity was correlated with reduced CCVD mortality risk (aHR=0.72, p=0.035). Overall, a high BMI and TyG index increase mortality risk, particularly in older adults (**Supplementary Table S7**). The statistical models indicate reliable estimates and no significant concerns about multicollinearity affecting the interpretation of the results (**Supplementary Table S8**).

## Discussion

Our study investigated the individual and joint effects of overweight/obesity and the triglyceride−glucose (TyG) index on mortality risk in T2DM patients. Among the participants, 1,665 deaths were recorded, with higher mortality observed in the lowest TyG quartile (Q1) than in the other quartiles (Q2-Q4). The deceased participants tended to be older and had greater incidences of stroke, hypertension, and fatty liver disease, along with lower kidney function. Overweight individuals presented a reduced risk of mortality, whereas obesity presented a less significant effect. A high TyG index was also linked to a lower mortality risk, with those in the highest quartile having a 16% lower risk for all-cause mortality and CCVD mortality, and the risk of overweight mortality was reduced by 31%. Individuals with a higher TyG index were similarly associated with reduced CCVD mortality. The combination of overweight status and elevated TyG was associated with the lowest all-cause mortality risk. No significant interaction was observed between BMI and the TyG index in patients with type 2 diabetes mellitus (T2DM). While we found that overweight and obese people with T2DM have a lower risk of death, this result should be viewed as the “obesity paradox.” This paradox may result from methodological artefacts such as reverse causality, residual confounding, or more specifically, collider stratification bias. In the joint-effect analyses, the TyG index was dichotomized using the median value to enhance the interpretability of the results. This approach was adopted due to the absence of a clinically validated TyG threshold for mortality risk stratification in patients with T2DM. The median-based cutoff represents a methodologically sound and practical solution under these circumstances. Importantly, analyses treating the TyG index as both a continuous and a categorical variable yielded consistent correlation patterns, suggesting that the observed associations are not merely artefacts of the chosen categorization scale but reflect genuine relationships between insulin resistance and mortality risk.

Several limitations of this study are inherent to its observational design. Although we adjusted for a wide range of demographic, clinical, and metabolic confounders and performed multiple sensitivity analyses, the possibility of residual confounding cannot be entirely ruled out. Specific biases, such as reverse causation and collider stratification, may partly explain the observed associations between elevated BMI, a higher TyG index, and increased mortality risk, particularly in older adults. While advanced causal inference approaches, such as marginal structural models or target trial emulation, could have strengthened causal interpretation, their application was constrained by the lack of time-varying exposure measurements and detailed longitudinal treatment data. Therefore, our findings should be interpreted as associative rather than causal. Future studies incorporating repeated measures of BMI, TyG index, and medication use, along with formal causal modelling, are warranted to validate these relationships and inform clinical practice. Our findings are consistent with the “obesity paradox”, which explores BMI within the overweight category and indicates a lower mortality risk among elderly individuals who have chronic conditions such as T2DM (21, 22). Compared with normal-weight individuals, overweight individuals may have some mortality benefits when ageing factors are considered (23, 24). These findings are crucial for considering the complex interactions between BMI categories and mortality risk, while accounting for various confounding factors. A similar conclusion states that changes in risk estimates depend primarily on adjustments for comorbidities (25). The associations were consistent across multiple models adjusted for demographic and clinical factors. The TyG index has been accepted as a significant predictor of insulin resistance in the development and mortality of T2DM (10, 11, 26). This study aligns with our observations that controlled TyG index values are associated with increased all-cause and CCVD mortality risk among the T2DM population, thereby reducing mortality. This trend consistently remained even after adjusting for factors (including age, sex, and other comorbidities). This consistent link suggested that obesity indices and insulin resistance measures should be considered when evaluating mortality risk in T2DM patients. A previous study revealed that compared with tests of individual components, combined BMI and TyG index assessments are associated with better patient outcomes (27). Additionally, the joint effects analysis of BMI and TyG revealed significant differences among T2DM patients with respect to mortality risk. Overweight or obese individuals with a high TyG index had a reduced mortality risk, whereas normal-weight individuals with a low TyG index had a moderately increased mortality risk in T2DM patients. This outcome supports the findings of various studies indicating that metabolic health indicators such as TyG may help predict health outcomes other than BMI categories (28, 29). Consequently, the findings of this study highlight the need for researchers to conduct further causal inference methods research to provide details on whether healthcare providers to acknowledge that they need to incorporate both BMI and metabolic markers such as TyG to evaluate the existing risk factors among T2DM patients. Furthermore, adult age (≥ 60 years) indicates increased mortality risk in individuals with T2DM, especially individuals who are overweight and have a high TyG index. This trend is particularly prevalent in individuals who are overweight and have high TyG levels. This increased risk may be due to a greater established prevalence of physiological factors, including a decline in insulin resistance and increased comorbidities in older patients (30, 31). Obesity and metabolic dysregulation might be more challenging for aging adults than for young people because later age is associated with health worsened by a decline in muscle mass and function (32, 33). The U-shaped curve observed, with mortality risk decreasing as BMI increased for extremely high BMI values, indicates support for a previous study on the obesity paradox, where moderate obesity improves outcomes but excessive weight does not confer additional benefits (34, 35). Although obesity provides some mortality benefits, the protective effect is reduced compared with that in overweight patients; consistently, supporting moderate weight gain combined with metabolic control is the optimal strategy (36). These findings imply that a “one-size-fits-all” approach to weight management in diabetes patients may not be optimal (37, 38). Clinicians might consider individualized targets for BMI and TyG on the basis of patient demographics and comorbidities to optimize survival outcomes. This study suggests that moderate adiposity combined with controlled insulin resistance (as indicated by TyG) may provide survival benefits for patients with type 2 diabetes. These findings dispel traditional concepts, according to which decreased BMI always has an improved impact on diabetic patients (39). The major strength of our study is that it has a relatively large sample size of 15,796 T2DM patients, which increases the credibility of the findings. Hence, in addition to BMI and the TyG index, this retrospective cohort study incorporates separate and joint impacts of individual components on mortality. Additionally, various confounders such as age, sex, hypertension, coronary heart disease, stroke, fatty liver disease, smoking and drinking, HbA1c, HDK-C, LDD-C, and eGFR were accurately controlled, which further established the accuracy of the conclusions. Furthermore, it explores subgroup analyses to obtain different and improved results per age group and follow-up interval. This counterintuitive phenomenon may reflect not a true protective effect but rather statistical artifacts such as reverse causality, residual confounding, or particularly collider stratification bias (40, 41).

### Limitations

Since all participants in this study had T2DM, conditioning could have resulted in collider bias, potentially causing noncausal relationships between BMI and mortality. Several limitations are acknowledged in our study. First, the study is an observational study that cannot identify causal associations between BMI and the TyG index and mortality risk. Despite extensive multivariable adjustments aimed at minimizing potential confounding, the possibility of residual confounding cannot be entirely ruled out. A particular methodological concern is reverse causation, whereby pre-existing illness, especially severe or systemic disease, may lead to weight loss, rather than low body weight itself contributing to elevated mortality. This form of bias can arise when undiagnosed or preclinical conditions cause changes in adiposity before baseline assessment, potentially distorting the true association between weight status and survival outcomes (42, 43). Although analytical strategies, such as excluding early mortality or participants with known chronic diseases, were employed to mitigate this issue, the complete elimination of reverse causality remains challenging in observational studies of body weight and mortality (43, 44). To minimize the potential for reverse causation, sensitivity analyses excluded patients who died within the first two years of follow-up. Second, our data source was electronic medical records; the sample population was obtained from two hospitals and primarily consisted of patients who received inpatient or specialist-based care. Compared to community-diagnosed patients, this hospital-based cohort is likely to experience longer disease duration, greater comorbidity burden, and higher healthcare utilization, which may limit the generalizability of our findings to primary care or general population settings. Therefore, caution is warranted when extrapolating these results to broader T2DM populations. Another notable limitation is the exclusion of underweight individuals (BMI<18.5 kg/m^2^). Although underweight has been consistently associated with elevated mortality in T2DM, disentangling causal effects from reverse causation, where weight loss results from underlying illness, remains challenging in observational studies (22). Third, the inclusion of a small underweight subgroup without longitudinal weight history could have introduced bias into the obesity paradox estimates and joint-effect analyses. Future studies incorporating larger underweight populations, repeated BMI measurements, and detailed metabolic profiles are needed to clarify the independent and combined effects of low BMI and insulin resistance on mortality risk. Fourth, some relevant comorbidities, such as familial dyslipidemia and metabolic dysfunction–associated steatohepatitis (MASD), were not available in the dataset, which may influence the interpretation of TyG-related findings. Fifth, T2DM diagnoses were derived from electronic medical records based on clinicians’ assessments, and BMI and the TyG index were used as surrogate markers rather than comprehensive indicators of metabolic health. Sixth, we lacked data on weight history and unintentional weight loss, which may influence mortality outcomes. Seventh, BMI and TyG were measured only at baseline, with accounting for potential changes over time. Further research employing a time-varying/longitudinal model should be conducted to investigate the effect of dynamic variations in adiposity and insulin resistance on mortality at various stages of disease development. Eighth, the analysis included various crucial confounding variables, but we cannot rule out the influence of unknown confounders on our study results. Thus, our fine−gray model analysis managed competing risks in mortality outcomes, but we did not exhaustively evaluate alternative biases due to competing risks, which might skew mortality estimates, especially in distinct patient groups. Thus, our findings may not justify changes in clinical guidelines until they are validated by randomized controlled trials or causal inference methods such as marginal structural models. Further research is needed to refine the clinical management guidelines for BMI and the TyG index among T2DM patients.

## Conclusion

This retrospective cohort study evaluated the relationships among overweight/obesity, the TyG index, and mortality risk in patients with type 2 diabetes mellitus (T2DM). Our findings revealed multiple associations between BMI and the TyG index when mortality risk in T2DM patients was compared. This is particularly true among older adults. However, individuals with higher BMIs and TyG indices had lower mortality risk in T2DM. These observations do not imply causality or confirm prognostic value. Further research employing causal inference methods should be applied to examine the causal links between these factors for the use of clinical practice.

## Data Availability

The data analyzed in this study is subject to the following licenses/restrictions: The data supporting this study are derived from electronic medical records and contain sensitive patient information. Due to institutional privacy policies and ethical considerations, the dataset is not publicly available. However, de-identified data may be made available from the corresponding author upon reasonable request and with appropriate institutional approval. Requests to access these datasets should be directed to Pro. Guangyun Mao, mgy@wmu.edu.cn.

## Supplementary material

Supplementary Table 1 Comparison before and after multiple imputation of missing variables. HDL-C, high density lipoprotein cholesterol; LDL-C, low density lipoprotein cholesterol; SCr, serum creatinine; HbA1c, glycosylated hemoglobin A1c; SMD, standardized mean difference.

Supplementary Table 2 Individual effects of BMI and the TyG index on the risk of mortality in patients with type 2 diabetes (after excluding patients who died within 2 years of follow-up). BMI, body mass index; TyG, triglyceride−glucose; HR, hazard ratio; CI, confidence interval; CCVD, cardio-cerebrovascular disease. Per-IQR, per interquartile range increase in TyG; Q1 - Q4, quartiles of TyG. Age, sex, TyG, hypertension, coronary heart disease, stroke, fatty liver disease, smoking, alcohol consumption, glycosylated hemoglobin A1c, high-density lipoprotein cholesterol, low-density lipoprotein cholesterol and estimated glomerular filtration rate were adjusted for in the associations between BMI and mortality. Age, sex, BMI, hypertension, coronary heart disease, stroke, fatty liver disease, smoking, alcohol consumption, glycosylated hemoglobin A1C, high-density lipoprotein cholesterol, low-density lipoprotein cholesterol and estimated glomerular filtration rate were adjusted for the associations between TyG and mortality.

Supplementary Table 3 Joint effects of BMI and the TyG index on the risk of mortality in patients with type 2 diabetes (after excluding patients who died within 2 years of follow-up). BMI, body mass index; TyG, triglyceride−glucose; HR, hazard ratio; CI, confidence interval; CCVD, cardio-cerebrovascular disease. Adjusted for age, sex, hypertension, coronary heart disease, stroke, fatty liver disease, smoking, drinking, glycosylated hemoglobin A1c, high-density lipoprotein cholesterol, low-density lipoprotein cholesterol, and estimated glomerular filtration rate.

Supplementary Table 4 Individual effects of BMI and the TyG index on the risk of CCVD mortality in patients with type 2 diabetes (Fine−Gray model). BMI, body mass index; TyG, triglyceride−glucose; HR, hazard ratio; CI, confidence interval; CCVD, cardio-cerebrovascular disease. Per-IQR, per interquartile range increase in TyG; Q1 - Q4, quartiles of TyG Age, sex, TyG, hypertension, coronary heart disease, stroke, fatty liver disease, smoking, drinking, glycosylated hemoglobin A1c, high-density lipoprotein cholesterol, low-density lipoprotein cholesterol, and estimated glomerular filtration rate were adjusted for in the associations between BMI and mortality. Age, sex, BMI, hypertension, coronary heart disease, stroke, fatty liver disease, smoking, drinking, glycosylated hemoglobin A1c, high-density lipoprotein cholesterol, low-density lipoprotein cholesterol, and estimated glomerular filtration rate were adjusted for in the associations between TyG and mortality.

Supplementary Table 5 Joint effects of BMI and the TyG index on the risk of CCVD mortality in patients with type 2 diabetes (Fine−Gray model). BMI, body mass index; TyG, triglyceride−glucose; HR, hazard ratio; CI, confidence interval; CCVD, cardio-cerebrovascular disease. Adjusted for age, sex, hypertension, coronary heart disease, stroke, fatty liver disease, smoking, drinking, glycosylated hemoglobin A1c, high-density lipoprotein cholesterol, low-density lipoprotein cholesterol, and estimated glomerular filtration rate.

Supplementary Table 6 Individual effects of BMI and the TyG index on the risk of mortality in patients with type 2 diabetes (stratified by age). BMI, body mass index; TyG, triglyceride−glucose; HR, hazard ratio; CI, confidence interval; CCVD, cardio-cerebrovascular disease. Per-IQR, per interquartile range increase in TyG; Q1 - Q4, quartiles of TyG. The associations between BMI and mortality were adjusted for sex, TyG, hypertension, coronary heart disease, stroke, fatty liver disease, smoking, drinking, glycosylated hemoglobin A1c, high-density lipoprotein cholesterol, low-density lipoprotein cholesterol, and estimated glomerular filtration rate. The associations between the TyG score and mortality were adjusted for sex, BMI, hypertension, coronary heart disease, stroke, fatty liver disease, smoking, drinking, glycosylated hemoglobin A1c, high-density lipoprotein cholesterol, low-density lipoprotein cholesterol, and estimated glomerular filtration rate in the association between TyG score and mortality.

Supplementary Table 7 Joint effects of BMI and the TyG index on the risk of mortality in patients with type 2 diabetes (stratified by age). BMI, body mass index; TyG, triglyceride−glucose; HR, hazard ratio; CI, confidence interval; CCVD, cardio-cerebrovascular disease. The model was adjusted for sex, hypertension, coronary heart disease, stroke, fatty liver disease, smoking, drinking, glycosylated hemoglobin A1c, high-density lipoprotein cholesterol, low-density lipoprotein cholesterol, and estimated glomerular filtration rate.

Supplementary Table 8 Variance inflation factors (VIFs) for variables in the adjusted model. CHD, coronary heart disease; HDL-C, high-density lipoprotein cholesterol; LDL-C, low-density lipoprotein cholesterol; HbA1c, glycosylated hemoglobin A1c; eGFR, estimated glomerular filtration rate.
