# Supplementary material for: Individual and joint effects of overweight/obesity and the triglyceride−glucose index on mortality risk in type 2 diabetic patients: a retrospective cohort study in China
Source: Front Endocrinol (Lausanne). 2026 Feb 6;17:1652682. doi: 10.3389/fendo.2026.1652682 (PMC12921575; doi:10.3389/fendo.2026.1652682)
Supplement: Supplementary Table 1 — Comparison before and after multiple imputation of missing variables. HDL-C, high density lipoprotein cholesterol; LDL-C, low density lipoprotein cholesterol; SCr, serum creatinine; HbA1c, glycosylated hemoglobin A1c; SMD, standardized mean difference. [file DataSheet1.pdf]

Table1.Baseline demographic and clinical characteristics of patients with type 2 diabetes according to all-cause mortality.

Variables	Overall (n=15796)	Alive (n=14131)	Dead (n=1665)	*P*-value	SMD
Sex				0.014	0.064
Male	8433 (53.4)	7497 (53.1)	936 (56.2)		
Female	7363 (46.6)	6634 (46.9)	729 (43.8)		
Smoke				0.893	0.003
No	11828 (74.9)	10579 (74.9)	1249 (75.0)		
Yes	3968 (25.1)	3552 (25.1)	416 (25.0)		
Drink				0.050	0.052
No	12367 (78.6)	11034 (78.4)	1333 (80.4)		
Yes	3372 (21.4)	3048 (21.6)	324 (19.6)		
CHD				0.001	0.083
No	5192 (32.9)	4586 (32.5)	606 (36.4)		
Yes	10604 (67.1)	9545 (67.5)	1059 (63.6)		
Stroke				<0.001	0.266
No	12620 (79.9)	11459 (81.1)	1161 (69.7)		
Yes	3176 (20.1)	2672 (18.9)	504 (30.3)		
Hypertension				<0.001	0.201
No	10774 (68.2)	9504 (67.3)	1270 (76.3)		
Yes	5022 (31.2)	4627 (32.7)	395 (23.7)		
Fatty liver disease				<0.001	0.328
No	6564 (41.6)	6103 (43.2)	461 (27.7)		
Yes	9232 (58.5)	8028 (56.8)	1204 (72.3)		
Age, years	65.0 (57.0, 73.0)	64.0 (57.0, 72.0)	74.0 (65.0, 80.0)	<0.001	0.798
HDL-C, mmol/L	1.0 (0.9, 1.2)	1.0 (0.9, 1.2)	0.9 (0.8, 1.1)	<0.001	0.157
LDL-C, mmol/L	2.5 (1.9, 3.2)	2.5 (1.9, 3.2)	2.4 (1.8, 3.1)	<0.001	0.108
SCr, µmol/L	64.7 (53.5, 79.0)	63.7 (53.0, 77.5)	73.9 (60.1, 93.1)	<0.001	0.471
HbA1c, %	7.8 (6.9, 8.8)	7.8 (6.9, 8.8)	7.9 (7.0, 8.9)	0.006	0.062
FPG, mmol/L	6.9 (5.7, 8.8)	6.9 (5.7, 8.8)	7.1 (5.6, 9.2)	0.095	0.064
TG, mmol/L	1.5 (1.1, 2.2)	1.6 (1.1, 2.2)	1.4 (1.0, 2.0)	<0.001	0.136
eGFR (mL/min per 1.73 m,%)	94.9 (76.6, 115.1)	96.8 (78.6, 116.5)	79.8 (62.8, 99.1)	<0.001	0.521

Normally distributed continuous variables were described as means ± standard deviation, and continuous variables without a normal distribution were presented as medians (1st quartile, 3rd quartile). Categorical variables were presented as numbers (percentages).

CHD, coronary heart disease; HDL-C, high density lipoprotein cholesterol; LDL-C, low density lipoprotein cholesterol; SCr, serum creatinine; HbA1c, glycosylated hemoglobin A1c; FPG, fasting plasma glucose; TG, triglyceride; eGFR, estimated glomerular filtration rate; SMD, standardized mean difference.

Table2. Individual effects of BMI and TyG on the risk of mortality in patients with type 2 diabetes.

			Crude		Adjusted	
Variable	N	Death Number (%)	HR (95% CI)	*P*-value	HR (95% CI)	*P*-value
All-cause mortality						
BMI, kg/m^2						
18.5-23.9 (Normal weight)	7152	882 (12.3)	Reference		Reference	
24.0-27.9 (Overweight)	6410	568 (8.9)	0.72 (0.65, 0.80)	<0.001	0.73 (0.65, 0.81)	<0.001
≥28.0 (Obesity)	2234	215 (9.6)	0.79 (0.68, 0.91)	0.002	0.86 (0.74, 1.00)	0.056
Per kg/m^2			0.96 (0.94, 0.97)	<0.001	0.97 (0.95, 0.98)	<0.001
TyG						
Q1 (7.46-8.67)	3949	481 (12.2)	Reference		Reference	
Q2 (8.67-9.08)	3949	414 (10.5)	0.86 (0.76, 0.99)	0.030	0.93 (0.81, 1.06)	0.262
Q3 (9.08-9.52)	3949	389 (9.9)	0.80 (0.70, 0.91)	0.001	0.85 (0.74, 0.98)	0.021
Q4 (9.52-11.1)	3949	381 (9.7)	0.78 (0.68, 0.89)	<0.001	0.84 (0.72, 0.97)	0.015
Per IQR=0.85			0.88 (0.83, 0.94)	<0.001	0.91 (0.85, 0.97)	0.005
CCVD mortality						
BMI, kg/m^2						
18.5-23.9 (Normal weight)	7152	378 (5.3)	Reference		Reference	
24.0-27.9 (Overweight)	6410	240 (3.7)	0.71 (0.60, 0.84)	<0.001	0.69 (0.58, 0.81)	<0.001
≥28.0 (Obesity)	2234	111 (5.0)	0.95 (0.77, 1.17)	0.618	1.03 (0.83, 1.28)	0.789
Per kg/m^2			0.98 (0.96, 1.00)	0.060	0.99 (0.96, 1.01)	0.201
TyG						
Q1 (7.46-8.67)	3949	205 (5.2)	Reference		Reference	
Q2 (8.67-9.08)	3949	195 (4.9)	0.96 (0.79, 1.16)	0.650	1.00 (0.81, 1.22)	0.972
Q3 (9.08-9.52)	3949	160 (4.1)	0.77 (0.63, 0.95)	0.013	0.77 (0.62, 0.96)	0.018
Q4 (9.52-11.1)	3949	169 (4.3)	0.81 (0.66, 0.99)	0.041	0.81 (0.65, 1.01)	0.064
Per IQR=0.85			0.88 (0.80, 0.97)	0.007	0.87 (0.79, 0.96)	0.007

BMI, body mass index; TyG, triglyceride-glucose; HR, hazard ratio; CI, confidence interval, CCVD, cardio-cerebrovascular diseases.
Per-IQR, per interquartile range increase of TyG; Q1 - Q4, quartiles of TyG.
Adjusted for age, sex,TyG, hypertension, coronary heart disease, stroke, fatty liver disease, smoke, drink, glycosylated hemoglobin A1c, high density lipoprotein cholesterol, low density lipoprotein cholesterol and estimated glomerular filtration rate in the association between BMI and mortality.
Adjusted for age, sex, BMI, hypertension, coronary heart disease, stroke, fatty liver disease, smoke, drink, glycosylated hemoglobin A1c, high density lipoprotein cholesterol, low density lipoprotein cholesterol and estimated glomerular filtration rate in the association between TyG and mortality.

Table3. Joint effects of BMI and TyG on the risk of mortality in patients with type 2 diabetes.

BMI, kg/m^2	TyG			Crude		Adjusted	
	≥9.08	N	Dead Number (%)	HR (95% CI)	*P*-value	HR (95% CI)	*P*-value
All-cause mortality							
18.5-23.9 (Normal weight)	No	3939	499 (12.7)	Reference		Reference	
18.5-23.9 (Normal weight)	Yes	3213	383 (11.9)	0.90 (0.79, 1.03)	0.141	0.89 (0.77, 1.02)	0.095
24.0-27.9 (Overweight)	No	3005	290 (9.7)	0.76 (0.65, 0.87)	<0.001	0.74 (0.64, 0.85)	<0.001
24.0-27.9 (Overweight)	Yes	3405	278 (8.2)	0.63 (0.55, 0.73)	<0.001	0.63 (0.54, 0.73)	<0.001
≥28.0 (Obesity)	No	954	106 (11.1)	0.85 (0.69, 1.05)	0.138	0.86 (0.70, 1.06)	0.166
≥28.0 (Obesity)	Yes	1280	109 (8.52)	0.67 (0.55, 0.83)	<0.001	0.76 (0.62, 0.94)	0.012
Interaction TyG Overweight				1.08 (0.75, 1.14)	0.459	1.04 (0.78, 1.19)	0.711
Interaction TyG Obestiy				1.15 (0.65, 1.18)	0.373	1.00 (0.74, 1.34)	0.974
$RERI_{overweight}$ (95% CI)				-0.03 (-0.20, 0.14)		0.00 (-0.17, 0.17)	
$RERI_{obesity}$ (95% CI)				-0.08 (-0.33, 0.16)		0.01 (-0.24, 0.26)	
CCVD mortality							
18.5-23.9 (Normal weight)	No	3939	220 (5.6)	Reference		Reference	
18.5-23.9 (Normal weight)	Yes	3213	158 (4.9)	0.85 (0.69, 1.04)	0.107	0.78 (0.63, 0.97)	0.023
24.0-27.9 (Overweight)	No	3005	124 (4.1)	0.73 (0.59, 0.91)	0.006	0.69 (0.55, 0.86)	0.001
24.0-27.9 (Overweight)	Yes	3405	116 (3.4)	0.60 (0.48, 0.75)	<0.001	0.54 (0.43, 0.69)	<0.001
≥28.0 (Obesity)	No	954	56 (5.9)	1.02 (0.76, 1.37)	0.881	1.01 (0.75, 1.36)	0.952
≥28.0 (Obesity)	Yes	1280	55 (4.3)	0.77 (0.57, 1.04)	0.084	0.82 (0.61, 1.12)	0.213
Interaction TyG Overweight				1.04 (0.70, 1.33)	0.822	0.99 (0.73, 1.40)	0.942
Interaction TyG Obestiy				1.12 (0.58, 1.36)	0.598	0.96 (0.68, 1.60)	0.840
$RERI_{overweight}$ (95% CI)				0.02 (-0.23, 0.27)		0.07 (-0.16, 0.31)	
$RERI_{obesity}$ (95% CI)				-0.10 (-0.49, 0.29)		0.03 (-0.35, 0.42)	

BMI, body mass index; TyG, triglyceride-glucose; HR, hazard ratio; CI, confidence interval, CCVD, cardio-cerebrovascular diseases; RERI, relative excess risk due to interaction.

Adjusted for age, sex, hypertension, coronary heart disease, stroke, fatty liver disease, smoke, drink, glycosylated hemoglobin A1c, high density lipoprotein cholesterol, low density lipoprotein cholesterol and estimated glomerular filtration rate.

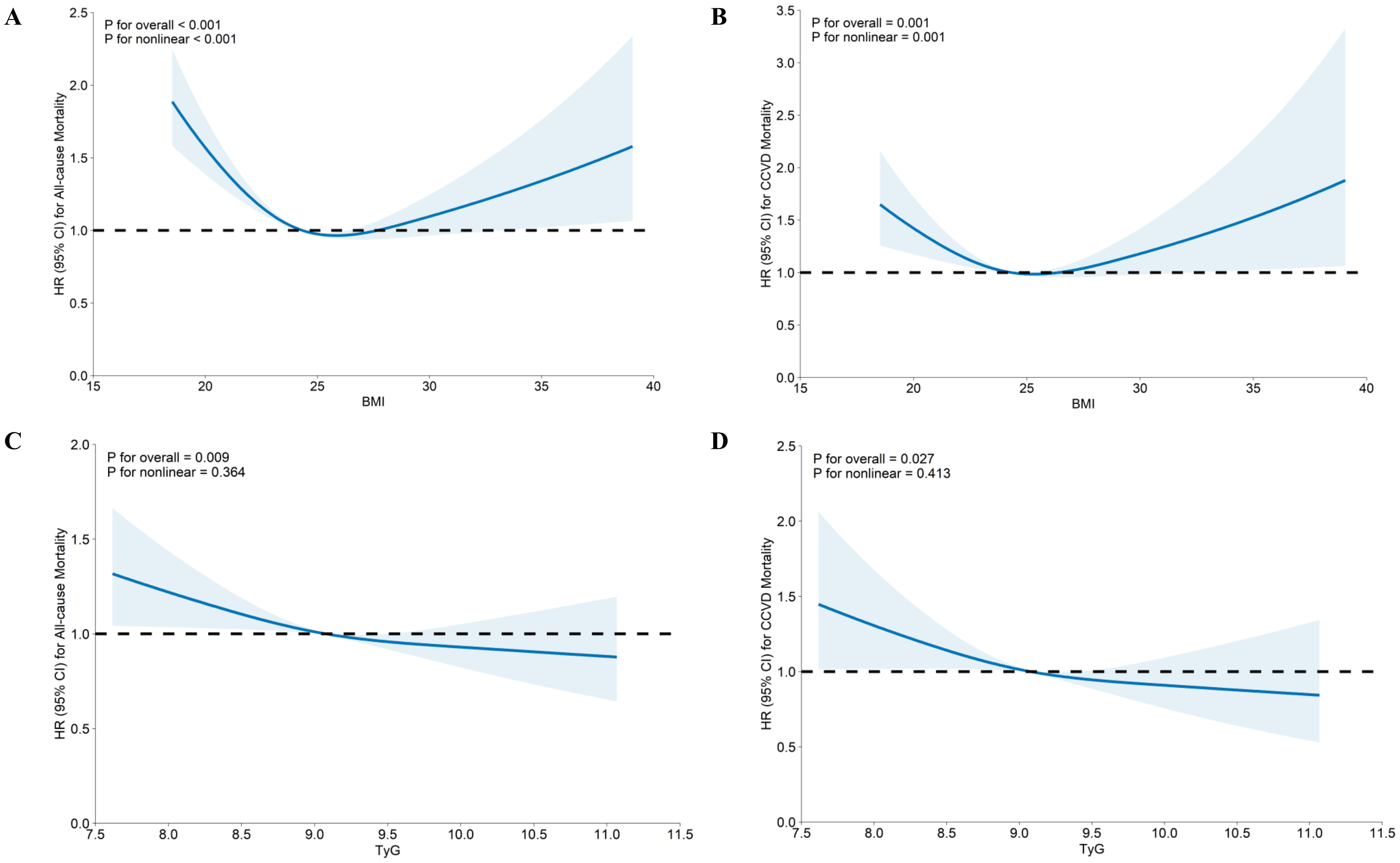

Figure1. Associations of BMI and TyG with the risk of all-cause mortality and CCVD mortality based on spline regression models.

BMI, body mass index; TyG, triglyceride-glucose; HR, hazard ratio; CI, confidence interval, CCVD, cardio-cerebrovascular diseases.

Adjusted for age, sex, TyG, hypertension, coronary heart disease, stroke, fatty liver disease, smoke, drink, glycosylated hemoglobin A1c, high density lipoprotein cholesterol, low density lipoprotein cholesterol and estimated glomerular filtration rate in the association between BMI and mortality.

Adjusted for age, sex, BMI, hypertension, coronary heart disease, stroke, fatty liver disease, smoke, drink, glycosylated hemoglobin A1c, high density lipoprotein cholesterol, low density lipoprotein cholesterol and estimated glomerular filtration rate in the association between TyG and mortality.

(A) BMI and all-cause mortality, (B) BMI and CCVD mortality; (C) TyG and all-cause mortality; (D) TyG and CCVD mortality.

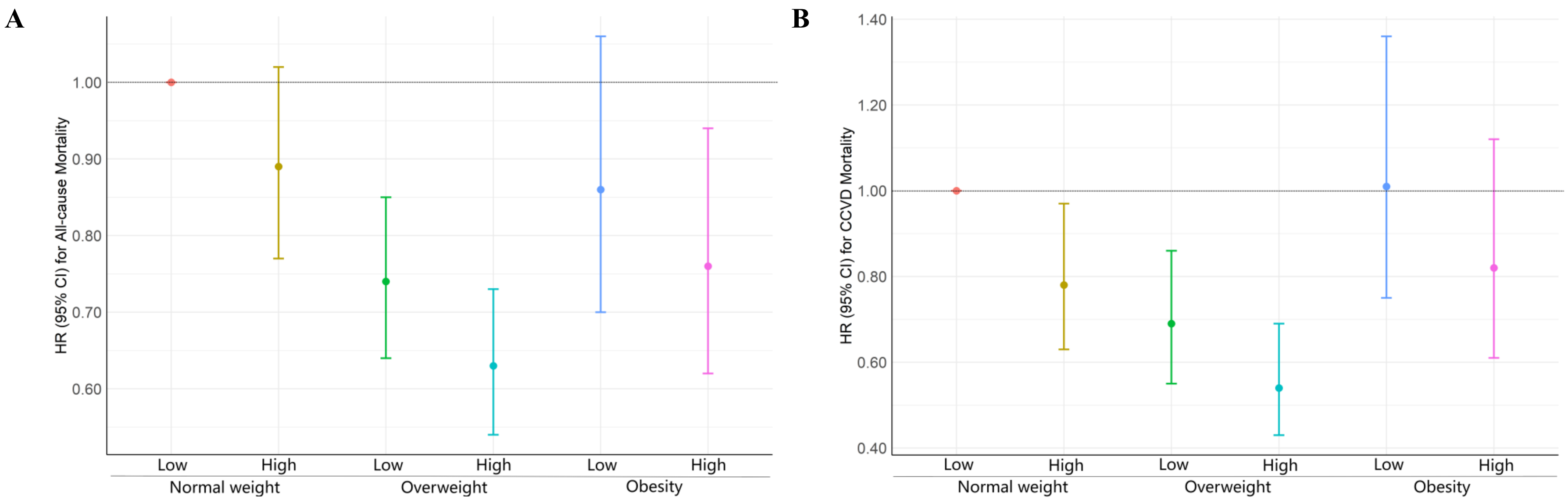

Figure2. Adjusted combined effects of BMI and TyG on the risk of all-cause mortality and CCVD mortality.

TyG, triglyceride-glucose; HR, hazard ratio; CI, confidence interval, CCVD, cardio-cerebrovascular diseases.

Adjusted for age, sex, body mass index, hypertension, coronary heart disease, stroke, fatty liver disease, smoke, drink, glycosylated hemoglobin A1c, high density lipoprotein cholesterol, low density lipoprotein cholesterol and estimated glomerular filtration rate.

Normal weight, overweight, and obesity categories indicating BMI are 18.5–23.9, 24.0–27.9 and ≥28 kg/m^2, respectively; Low indicates TyG < 9.08; High indicates TyG ≥ 9.08.

Supplementary Table S1. Comparison before and after multiple imputation of missing variables.

Variables	Before	After	*P-value*	SMD
HDL-C, mmol/L	1.0(0.8,1.2)	1.0(0.9,1.2)	0.102	0.001
LDL-C, mmol/L	2.5(1.9,3.2)	2.5(1.9,3.2)	0.840	0.005
SCr, µmol/L	64.0(53.0,78.7)	64.7(53.5,79.0)	0.097	0.006
HbA1c, %	7.6(6.7,9.0)	7.8(6.9,8.8)	<0.001	0.006

HDL-C, high density lipoprotein cholesterol; LDL-C, low density lipoprotein cholesterol; SCr, serum creatinine; HbA1c, glycosylated hemoglobin A1c; SMD, standardized mean difference.

Supplementary TableS2. Individual effects of BMI and TyG on the risk of mortality in patients with type 2 diabetes (after excluding patients who died within 2 years of follow-up).

			Crude		Adjusted	
Variable	N	Death Number (%)	HR (95% CI)	*P*-value	HR (95% CI)	*P*-value
All-cause mortality						
BMI, kg/m^2						
18.5-23.9 (Normal weight)	6753	483 (7.2)	Reference		Reference	
24.0-27.9 (Overweight)	6184	342 (5.5)	0.79 (0.69, 0.91)	0.001	0.79 (0.69, 0.91)	0.001
≥28.0 (Obesity)	2148	129 (6.0)	0.86 (0.71, 1.04)	0.129	0.93 (0.76, 1.13)	0.482
Per kg/m^2			0.98 (0.96, 0.99)	0.018	0.98 (0.96, 1.00)	0.115
TyG						
Q1 (7.53-8.68)	3771	264 (7.0)	Reference		Reference	
Q2 (8.68-9.08)	3771	243 (6.4)	0.94 (0.79, 1.12)	0.503	0.99 (0.83, 1.19)	0.946
Q3 (9.08-9.53)	3771	210 (5.6)	0.79 (0.66, 0.94)	0.010	0.81 (0.67, 0.98)	0.030
Q4 (9.53-11.1)	3772	237 (6.3)	0.89 (0.75, 1.06)	0.206	0.94 (0.77, 1.13)	0.496
Per IQR=0.85			0.95 (0.88, 1.03)	0.196	0.97 (0.89, 1.06)	0.454
CCVD mortality						
BMI, kg/m^2						
18.5-23.9 (Normal weight)	6753	208 (3.1)	Reference		Reference	
24.0-27.9 (Overweight)	6184	139 (2.3)	0.75 (0.60, 0.93)	0.008	0.72 (0.58, 0.89)	0.003
≥28.0 (Obesity)	2148	67 (3.1)	1.04 (0.79, 1.37)	0.785	1.10 (0.83, 1.46)	0.493
Per kg/m^2			1.00 (0.97, 1.03)	0.722	1.00 (0.97, 1.03)	0.936
TyG						
Q1 (7.53-8.68)	3771	106 (2.8)	Reference		Reference	
Q2 (8.68-9.08)	3771	118 (3.1)	1.14 (0.88, 1.48)	0.329	1.15 (0.88, 1.51)	0.305
Q3 (9.08-9.53)	3771	84 (2.2)	0.78 (0.59, 1.04)	0.094	0.74 (0.55, 1.00)	0.053
Q4 (9.53-11.1)	3772	106 (2.8)	0.99 (0.76, 1.30)	0.954	0.96 (0.71, 1.28)	0.761
Per IQR=0.85			0.99 (0.87, 1.11)	0.809	0.97 (0.85, 1.10)	0.593

BMI, body mass index; TyG, triglyceride-glucose; HR, hazard ratio; CI, confidence interval, CCVD, cardio-cerebrovascular diseases.

Per-IQR, per interquartile range increase of TyG; Q1 - Q4, quartiles of TyG.

Adjusted for age, sex,TyG, hypertension, coronary heart disease, stroke, fatty liver disease, smoke, drink, glycosylated hemoglobin A1c, high density lipoprotein cholesterol, low density lipoprotein cholesterol and estimated glomerular filtration rate in the association between BMI and mortality.

Adjusted for age, sex, BMI, hypert ension, coronary heart disease, stroke, fatty liver disease, smoke, drink, glycosylated hemoglobin A1c, high density lipoprotein cholesterol, low density lipoprotein cholesterol and estimated glomerular filtration rate in the association between TyG and mortality.

Supplementary TableS3. Joint effects of BMI and TyG on the risk of mortality in patients with type 2 diabetes (after excluding patients who died within 2 years of follow-up).

BMI, kg/m^2	TyG			Crude		Adjusted	
	≥9.08	N	Dead Number (%)	HR (95% CI)	*P*-value	HR (95% CI)	*P*-value
All-cause mortality							
18.5-23.9 (Normal weight)	No	3722	265 (7.1)	Reference		Reference	
18.5-23.9 (Normal weight)	Yes	3031	218 (7.2)	0.96 (0.80, 1.15)	0.636	0.93 (0.77, 1.12)	0.429
24.0-27.9 (Overweight)	No	2899	174 (6.0)	0.85 (0.70, 1.03)	0.098	0.83 (0.69, 1.01)	0.065
24.0-27.9 (Overweight)	Yes	3285	168 (5.1)	0.71 (0.59, 0.87)	0.001	0.70 (0.57, 0.85)	<0.001
≥28.0 (Obesity)	No	921	68 (7.4)	1.00 (0.77, 1.31)	0.995	1.01 (0.77, 1.32)	0.941
≥28.0 (Obesity)	Yes	1227	61 (5.0)	0.72 (0.54, 0.95)	0.019	0.79 (0.59, 1.05)	0.106
CCVD mortality							
18.5-23.9 (Normal weight)	No	3722	115 (3.1)	Reference		Reference	
18.5-23.9 (Normal weight)	Yes	3031	93 (3.1)	0.94 (0.71, 1.23)	0.644	0.85 (0.64, 1.12)	0.246
24.0-27.9 (Overweight)	No	2899	74 (2.6)	0.83 (0.62, 1.12)	0.22	0.79 (0.59, 1.06)	0.116
24.0-27.9 (Overweight)	Yes	3285	65 (2.0)	0.64 (0.47, 0.86)	0.003	0.56 (0.40, 0.76)	<0.001
≥28.0 (Obesity)	No	921	35 (3.8)	1.19 (0.81, 1.73)	0.372	1.17 (0.80, 1.72)	0.415
≥28.0 (Obesity)	Yes	1227	32 (2.6)	0.87 (0.59, 1.28)	0.474	0.89 (0.59, 1.32)	0.555

BMI, body mass index; TyG, triglyceride-glucose; HR, hazard ratio; CI, confidence interval, CCVD, cardio-cerebrovascular diseases.

Adjusted for age, sex, hypertension, coronary heart disease, stroke, fatty liver disease, smoke, drink, glycosylated hemoglobin A1c, high density lipoprotein cholesterol, low density lipoprotein cholesterol and estimated glomerular filtration rate.

Supplementary TableS4. Individual effects of BMI and TyG on the risk of CCVD mortality in patients with type 2 diabetes (Fine-Gray model).

			Crude		Adjusted	
Variable	N	Death Number (%)	HR (95% CI)	*P*-value	HR (95% CI)	*P*-value
BMI, kg/m^2						
18.5-23.9 (Normal weight)	7152	378 (5.3)	Reference		Reference	
24.0-27.9 (Overweight)	6410	240 (3.7)	0.72 (0.61, 0.85)	<0.001	0.7 (0.6, 0.83)	<0.001
≥28.0 (Obesity)	2234	111 (5.0)	0.96 (0.78, 1.19)	0.738	1.05 (0.85, 1.3)	0.633
Per kg/m^2			0.99 (0.96, 1.01)	0.373	0.98 (0.96, 1.01)	0.128
TyG						
Q1 (7.46-8.67)	3949	205 (5.2)	Reference		Reference	
Q2 (8.67-9.08)	3949	195 (4.9)	0.96 (0.79, 1.17)	0.722	1.02 (0.83, 1.25)	0.866
Q3 (9.08-9.52)	3949	160 (4.1)	0.78 (0.63, 0.95)	0.016	0.78 (0.63, 0.97)	0.026
Q4 (9.52-11.1)	3949	169 (4.3)	0.82 (0.67, 1)	0.056	0.83 (0.67, 1.04)	0.109
Per IQR=0.85			0.88 (0.81, 0.97)	0.010	0.88 (0.79, 0.97)	0.011

BMI, body mass index; TyG, triglyceride-glucose; HR, hazard ratio; CI, confidence interval, CCVD, cardio-cerebrovascular diseases.

Per-IQR, per interquartile range increase of TyG; Q1 - Q4, quartiles of TyG.

Adjusted for age, sex,TyG, hypertension, coronary heart disease, stroke, fatty liver disease, smoke, drink, glycosylated hemoglobin A1c, high density lipoprotein cholesterol, low density lipoprotein cholesterol and estimated glomerular filtration rate in the association between BMI and mortality.

Adjusted for age, sex, BMI, hypert ension, coronary heart disease, stroke, fatty liver disease, smoke, drink, glycosylated hemoglobin A1c, high density lipoprotein cholesterol, low density lipoprotein cholesterol and estimated glomerular filtration rate in the association between TyG and mortality.

Supplementary TableS5. Joint effects of BMI and TyG on the risk of CCVD mortality in patients with type 2 diabetes (Fine-Gray model).

BMI, kg/m^2	TyG			Crude		Adjusted	
	≥9.08	N	Dead Number (%)	HR (95% CI)	*P*-value	HR (95% CI)	*P*-value
18.5-23.9 (Normal weight)	No	3939	220 (5.6)	Reference		Reference	
18.5-23.9 (Normal weight)	Yes	3213	158 (4.9)	0.117	0.117	0.79 (0.64, 0.98)	0.03
24.0-27.9 (Overweight)	No	3005	124 (4.1)	0.008	0.008	0.7 (0.56, 0.87)	0.002
24.0-27.9 (Overweight)	Yes	3405	116 (3.4)	<0.001	<0.001	0.56 (0.45, 0.71)	<0.001
≥28.0 (Obesity)	No	954	56 (5.9)	0.785	0.785	1.04 (0.78, 1.4)	0.772
≥28.0 (Obesity)	Yes	1280	55 (4.3)	0.113	0.113	0.85 (0.63, 1.15)	0.281

BMI, body mass index; TyG, triglyceride-glucose; HR, hazard ratio; CI, confidence interval, CCVD, cardio-cerebrovascular diseases.

Adjusted for age, sex, hypertension, coronary heart disease, stroke, fatty liver disease, smoke, drink, glycosylated hemoglobin A1c, high density lipoprotein cholesterol, low density lipoprotein cholesterol and estimated glomerular filtration rate.

Supplementary TableS6. Individual effects of BMI and TyG on the risk of mortality in patients with type 2 diabetes (stratified by age).

			Crude		Adjusted	
Variable	N	Death Number (%)	HR (95% CI)	*P*-value	HR (95% CI)	*P*-value
Age < 60 years						
All-cause mortality						
BMI, kg/m^2						
18.5-23.9 (Normal weight)	2063	105 (5.1)	Reference		Reference	
24.0-27.9 (Overweight)	2027	63 (3.1)	0.61 (0.45, 0.83)	0.002	0.58 (0.42, 0.80)	0.001
≥28.0 (Obesity)	839	30 (3.6)	0.73 (0.49, 1.10)	0.132	0.77 (0.51, 1.17)	0.229
Per kg/m^2			0.95 (0.91, 0.99)	0.036	0.96 (0.91, 1.00)	0.060
TyG						
Q1 (7.63-8.79)	1232	56 (4.6)	Reference		Reference	
Q2 (8.79-9.22)	1232	48 (3.9)	0.89 (0.60, 1.30)	0.540	0.92 (0.62, 1.37)	0.691
Q3 (9.22-9.66)	1233	53 (4.3)	1.04 (0.71, 1.51)	0.854	1.15 (0.78, 1.70)	0.493
Q4 (9.66-11.1)	1232	41 (3.3)	0.79 (0.53, 1.18)	0.252	0.87 (0.56, 1.33)	0.507
Per IQR=0.87			0.94 (0.79, 1.13)	0.509	0.97 (0.80, 1.18)	0.782
CCVD mortality						
BMI, kg/m^2						
18.5-23.9 (Normal weight)	2063	32 (1.6)	Reference		Reference	
24.0-27.9 (Overweight)	2027	17 (0.8)	0.54 (0.30, 0.97)	0.040	0.47 (0.26, 0.87)	0.016
≥28.0 (Obesity)	839	16 (1.9)	1.29 (0.71, 2.34)	0.412	1.07 (0.58, 2.00)	0.824
Per kg/m^2			1.02 (0.95, 1.10)	0.605	1.00 (0.92, 1.08)	0.930
TyG						
Q1 (7.63-8.79)	1232	16 (1.3)	Reference		Reference	
Q2 (8.79-9.22)	1232	18 (1.5)	1.17 (0.60, 2.30)	0.647	1.00 (0.50, 2.00)	0.993
Q3 (9.22-9.66)	1233	13 (1.1)	0.90 (0.43, 1.88)	0.783	0.73 (0.34, 1.57)	0.423
Q4 (9.66-11.1)	1232	18 (1.5)	1.24 (0.63, 2.44)	0.530	0.81 (0.39, 1.70)	0.583
Per IQR=0.87			1.20 (0.88, 1.64)	0.248	0.99 (0.70, 1.39)	0.950
Age ≥ 60 years						
All-cause mortality						
BMI, kg/m^2						
18.5-23.9 (Normal weight)	7152	882 (12.3)	Reference		Reference	
24.0-27.9 (Overweight)	6410	568 (8.9)	0.72 (0.65, 0.80)	<0.001	0.71 (0.63, 0.79)	<0.001
≥28.0 (Obesity)	2234	215 (9.6)	0.79 (0.68, 0.91)	0.002	0.81 (0.69, 0.94)	0.005
Per kg/m^2			0.96 (0.94, 0.97)	<0.001	0.96 (0.94, 0.97)	<0.001
TyG						
Q1 (7.46-8.67)	3949	481 (12.2)	Reference		Reference	
Q2 (8.67-9.08)	3949	414 (10.5)	0.86 (0.76, 0.99)	0.030	0.91 (0.80, 1.04)	0.178
Q3 (9.08-9.52)	3949	389 (9.9)	0.80 (0.70, 0.91)	0.001	0.82 (0.71, 0.94)	0.005
Q4 (9.52-11.1)	3949	381 (9.7)	0.78 (0.68, 0.89)	<0.001	0.77 (0.67, 0.90)	0.001
Per IQR=0.85			0.88 (0.83, 0.94)	<0.001	0.88 (0.82, 0.94)	<0.001
CCVD mortality						
BMI, kg/m^2						
18.5-23.9 (Normal weight)	7152	378 (5.3)	Reference		Reference	
24.0-27.9 (Overweight)	6410	240 (3.7)	0.71 (0.60, 0.84)	<0.001	0.67 (0.57, 0.79)	<0.001
≥28.0 (Obesity)	2234	111 (5.0)	0.95 (0.77, 1.17)	0.618	0.96 (0.78, 1.19)	0.718
Per kg/m^2			0.98 (0.96, 1.00)	0.060	0.98 (0.96, 1.00)	0.056
TyG						
Q1 (7.46-8.67)	3949	205 (5.2)	Reference		Reference	
Q2 (8.67-9.08)	3949	195 (4.9)	0.96 (0.79, 1.16)	0.650	0.98 (0.80, 1.20)	0.870
Q3 (9.08-9.52)	3949	160 (4.1)	0.77 (0.63, 0.95)	0.013	0.74 (0.60, 0.92)	0.007
Q4 (9.52-11.1)	3949	169 (4.3)	0.81 (0.66, 0.99)	0.041	0.75 (0.60, 0.94)	0.011
Per IQR=0.85			0.88 (0.80, 0.97)	0.007	0.84 (0.76, 0.93)	<0.001

BMI, body mass index; TyG, triglyceride-glucose; HR, hazard ratio; CI, confidence interval, CCVD, cardio-cerebrovascular diseases.

Per-IQR, per interquartile range increase of TyG; Q1 - Q4, quartiles of TyG.

Adjusted for sex, TyG, hypertension, coronary heart disease, stroke, fatty liver disease, smoke, drink, glycosylated hemoglobin A1c, high density lipoprotein cholesterol, low density lipoprotein cholesterol and estimated glomerular filtration rate in the association between BMI and mortality.

Adjusted for sex, BMI, hypert ension, coronary heart disease, stroke, fatty liver disease, smoke, drink, glycosylated hemoglobin A1c, high density lipoprotein cholesterol, low density lipoprotein cholesterol and estimated glomerular filtration rate in the association between TyG and mortality.

Supplementary TableS7. Joint effects of BMI and TyG on the risk of mortality in patients with type 2 diabetes (stratified by age).

BMI, kg/m^2	TyG			Crude		Adjusted	
		N	Dead Number (%)	HR (95% CI)	*P*-value	HR (95% CI)	*P*-value
Age < 60 years	≥9.22						
All-cause mortality							
18.5-23.9 (Normal weight)	No	1175	57 (4.9)	Reference		Reference	
18.5-23.9 (Normal weight)	Yes	888	48 (5.4)	1.19 (0.81, 1.75)	0.373	1.21 (0.81, 1.79)	0.354
24.0-27.9 (Overweight)	No	948	33 (3.5)	0.72 (0.47, 1.10)	0.131	0.68 (0.44, 1.05)	0.085
24.0-27.9 (Overweight)	Yes	1079	30 (2.8)	0.60 (0.39, 0.94)	0.025	0.59 (0.37, 0.92)	0.021
≥28.0 (Obesity)	No	341	14 (4.1)	0.85 (0.48, 1.53)	0.594	0.83 (0.46, 1.50)	0.543
≥28.0 (Obesity)	Yes	498	16 (3.2)	0.74 (0.43, 1.29)	0.292	0.83 (0.47, 1.48)	0.530
CCVD mortality							
18.5-23.9 (Normal weight)	No	1175	17 (1.5)	Reference		Reference	
18.5-23.9 (Normal weight)	Yes	888	15 (1.7)	1.27 (0.63, 2.54)	0.506	0.96 (0.47, 1.96)	0.902
24.0-27.9 (Overweight)	No	948	10 (1.1)	0.73 (0.33, 1.59)	0.430	0.63 (0.28, 1.43)	0.268
24.0-27.9 (Overweight)	Yes	1079	7 (0.7)	0.48 (0.20, 1.15)	0.099	0.33 (0.13, 0.80)	0.015
≥28.0 (Obesity)	No	341	7 (2.1)	1.42 (0.59, 3.43)	0.434	1.19 (0.49, 2.92)	0.700
≥28.0 (Obesity)	Yes	498	9 (1.8)	1.43 (0.64, 3.21)	0.386	0.92 (0.39, 2.18)	0.850
Age ≥ 60 years	≥9.08						
All-cause mortality							
18.5-23.9 (Normal weight)	No	3939	499 (12.7)	Reference		Reference	
18.5-23.9 (Normal weight)	Yes	3213	383 (11.9)	0.90 (0.79, 1.03)	0.141	0.87 (0.76, 1.00)	0.043
24.0-27.9 (Overweight)	No	3005	290 (9.7)	0.76 (0.65, 0.87)	<0.001	0.73 (0.63, 0.84)	<0.001
24.0-27.9 (Overweight)	Yes	3405	278 (8.2)	0.63 (0.55, 0.73)	<0.001	0.58 (0.50, 0.68)	<0.001
≥28.0 (Obesity)	No	954	106 (11.1)	0.85 (0.69, 1.05)	0.138	0.83 (0.67, 1.03)	0.086
≥28.0 (Obesity)	Yes	1280	109 (8.5)	0.67 (0.55, 0.83)	<0.001	0.67 (0.54, 0.83)	<0.001
CCVD mortality							
18.5-23.9 (Normal weight)	No	3939	220 (5.6)	Reference		Reference	
18.5-23.9 (Normal weight)	Yes	3213	158 (4.9)	0.85 (0.69, 1.04)	0.107	0.76 (0.62, 0.95)	0.013
24.0-27.9 (Overweight)	No	3005	124 (4.1)	0.73 (0.59, 0.91)	0.006	0.68 (0.54, 0.85)	0.001
24.0-27.9 (Overweight)	Yes	3405	116 (3.4)	0.60 (0.48, 0.75)	<0.001	0.51 (0.40, 0.64)	<0.001
≥28.0 (Obesity)	No	954	56 (5.9)	1.02 (0.76, 1.37)	0.881	0.98 (0.73, 1.31)	0.874
≥28.0 (Obesity)	Yes	1280	55 (4.3)	0.77 (0.57, 1.04)	0.084	0.72 (0.53, 0.98)	0.035

BMI, body mass index; TyG, triglyceride-glucose; HR, hazard ratio; CI, confidence interval, CCVD, cardio-cerebrovascular diseases.
Adjusted for sex, hypertension, coronary heart disease, stroke, fatty liver disease, smoke, drink, glycosylated hemoglobin A1c, high density lipoprotein cholesterol, low density lipoprotein cholesterol and estimated glomerular filtration rate.

Supplementary TableS8.Variance inflation factors (VIFs) for variables in the adjusted model.

Variables	VIFs
Age, years	1.20
Sex	1.55
Smoke	1.58
Drink	1.38
CHD	1.18
Stroke	1.07
Hypertension	1.08
Fatty liver disease	1.14
HDL-C, mmol/L	1.17
LDL-C, mmol/L	1.12
HbA1c, %	1.07
eGFR, (mL/min per 1.73 m,%)	1.20

CHD, coronary heart disease; HDL-C, high density lipoprotein cholesterol; LDL-C, low density lipoprotein cholesterol; HbA1c, glycosylated hemoglobin A1c; eGFR, estimated glomerular filtration rate.
